# Beyond Targets: Measuring Better and Rebuilding Trust Comment on "Gaming New Zealand’s Emergency Department Target: How and Why Did It Vary Over Time and Between Organisations?"

**DOI:** 10.34172/ijhpm.2020.38

**Published:** 2020-03-11

**Authors:** Richard Hamblin, Carl Shuker

**Affiliations:** Health Quality Intelligence, Health Quality and Safety Commission, Wellington, New Zealand.

**Keywords:** Targets, Gaming, Performance Measurement, Performance Improvement

## Abstract

Tenbensel and colleagues identify that a target for emergency department (ED) stays in New Zealand met with gaming in response from local hospitals. The result is in line with studies in other jurisdictions. The enthusiasm for targets and tight performance measurement in some health systems reflects a lack of trust in professionals to do the right thing for altruistic reasons. However such measurement systems have failed to address this loss of trust and may, ironically, have worsened the situation. A more promising approach for both improving performance and restoring trust may depend upon collaboration and partnership between consumers, local providers, and central agencies in agreeing and tracking appropriate local responses to high level national goals rather than imposing tight, and potentially misleading measures from the centre.

A new paper by Tenbensel and colleagues^1^ has found for the first time evidence that gaming of New Zealand’s national emergency department (ED) wait time target of six hours was rife in at least four district health boards.

The authors found that, as in the United Kingdom, patients were discharged from ED to short stay units that existed only in name, the clock was stopped on patients who had not been admitted or discharged, and for some the clock was simply stopped to protect the institution’s performance.

New Zealand’s set of ten varied targets for healthcare performance were instituted in 2007. This set has evolved and mutated over time and changes in government since, before retirement of public reporting of performance against these targets in 2018.^2^

To date, study of the issue of gaming in response to targets within the public sector has tended to show gaming occurs where either strong financial incentives were attached to the target^3^ or strong, formal threats to organisations and individuals existed – particularly for their continued employment or organisational viability. These were the regimes of so-called “targets and terror.”^4,5^ New Zealand did not embrace such a formal pay-for-performance structure, and while targets were incentivised through publication and strong informal pressure on management, New Zealand’s district health boards retained considerably greater autonomy than National Health Service (NHS) trusts under the United Kingdom’s target regime of the early 2000s. In spite of this, Tenbensel’s paper suggests that even the weaker incentives of New Zealand’s regime were sufficient to encourage gaming behaviour.

The target for ED wait times has operated consistently throughout and is the most studied of New Zealand’s target regime, and is the target, after that for immunisations, perhaps viewed most positively by staff and the public.^6-8^ These new findings that gaming of performance was widespread provide a useful counterpoint and context to how we understand the effects of targets on provider behaviour.

## What’s the Point of Targets?

So, is there any point in using targets in health systems? Their negative and distortionary effects and unintended consequences have been identified and are increasingly studied, if little known to the wider public. Yet understanding how a health system is performing is essential to ensure its quality; and the studies identifying perverse effects of target regimes also recognised the genuine improvements they engendered. Further, making information about quality available to the users and funders of public health systems seems a moral imperative, and one increasingly expected as a matter of course.

How can we resolve these tensions?

## The Letter Versus the Spirit

To answer this question some consideration of both targets themselves, their strengths and limitations, and the context in which they were introduced, is helpful.

It is vital to distinguish between a measure and a target. Measuring the distribution of time spent inside EDs is essential to running these safely, effectively and efficiently. However, an associated target is not the measure itself but an externally imposed constraint upon the system which uses the measure to promote a particular aim.

Herein lies the first limitation of targets. Targets are only rarely a direct expression of the aim they are promoting – the aim of the ED target is to prevent inappropriately long waits in ED, not give everyone a six-hour wait there. Thus, the target measure is often only a proxy which has, hitherto, been associated with the aim.

The trouble with fixating on proxies is summed up by what is known as “Goodhart’s Law,” named for the economist Charles Goodhart. In plain English this states that “once a measure becomes a target it ceases to be a good measure.”^9^ Or, more formally, the relationship between the measure and the aim breaks down once the achievement of the measure, rather than the aim itself, becomes the focus of those charged with achieving it.

Yet, as the paper demonstrates, targets tend to stimulate actions that produce progress towards the underlying aim, at least at first. The phenomenon of gaming associated with targets might best be described as “gilding the lily” of initial, genuine improvement, as seen in both ED waits and ambulance response times in the United Kingdom.^10,11^

Here too, falsification of data or the arrangement of services to meet the letter but not spirit of the target has been shown to produce telltale signs of “management to measure” – the distributional discontinuities and terminal digit preference bias (opportunistic rounding) Tenbesel and colleagues identify, among other evidence.

It is therefore attractive to seek a way of keeping the benefits of targets while limiting the opportunity for gaming. This paper, like others before it^12^ promotes the value of independent validation of measures, and this has an intuitive technocratic appeal. However, this approach also has limitations. Data collection has costs; data collection about the collection of data (which is what validation amounts to) still more so.

## The Evolution of Professional Performance in Public Services

An alternative approach might be to think about what targets represent within public systems. To do this, it may help us to revisit two concepts, one half a century old and the other from the 1990s.

Michael Lipsky, in his concept of “street-level bureaucracy,”^13,14^ sketches a picture of front-line work that many health professionals would recognise: staff interact with citizens, operate under resource constraint, and have considerable independence in how they undertake their job. They have the potential to affect considerably the lives of those receiving their services, and yet face ambiguous expectations about job performance. Lipsky explicitly recognises that the unavailability of appropriate performance measures limits the ability of managers to control the application of policy at the ground level.

This need for control, and the use of targets as a mechanism to gain this, chimes with Le Grand’s 1997 insight into the changing perception of public servants.^15^ From being seen as “knights” acting altruistically for the public good, public servants (including healthcare professionals in a publicly funded health system) became “knaves,” primarily motivated by self-interest. What was necessary therefore was a mechanism to harness this tendency to act in self-interest.

The so-called New Public Management that emerged in the 1990s reflected this belief in the self-interested public servant. As originally conceived, rigorous monitoring of services would support market and quasi-market mechanisms that would drive improvement of services through the self-interest of the provider (incentives being protection of their service and thus budget, increased income, personal kudos and so forth).

Over time, as evidence emerged that publication of performance data was far more likely to change provider behaviour because of its potential to harm reputations than it was to stimulate a market of informed consumers voting with their feet,^16^ measurement and publication in and of itself became a central policy thrust.^17^

## The Loss of Trust

Yet this development has failed to address what is implicit in both Lipsky’s and Le Grand’s insights: a mutual loss of trust between central government, public services and, crucially, the public.

To counteract this loss of trust, quantitative measurement (apparently objective and precise) is given the task of restoring trust (“one version of the truth” or “a shared understanding of reality”) and targets are given the task of providing accountability: from public service agencies to central government, and from central government to the public.

But measures and targets have been unable to bear the weight placed upon them, and how they have been used may even have served to intensify the mistrust. In our view, this has happened for two reasons: the perception of imposition of measure and the response that this engenders; and over-interpretation of a limited range of measures.

If front-line services believe that targets have been imposed with little understanding of the mechanisms of giving care, what is clinically meaningful, or even what is most pressing and important in an area, they will lose trust in central government’s genuine commitment to actual (as opposed to apparent) performance of the service. This will be exacerbated when, for reasons of practicality, a small number of access measures are presented as an overarching judgement on the overall quality of a service. This imposition and misrepresentation reduces the credibility of the measures themselves and providers respond by what Lipsky describes as a “simplification”: doing what is necessary to hit the target as easily as possible in order to devote more resources to actual local priority. In this way, according to Bevan and Hood’s useful classification, ‘honest triers’ who do not attempt to spin or fiddle data in their favour become ‘reactive gamers’ who do.^4^ All incentives are to hit the target, and inevitably the threshold will arise when doing so means missing the point.

As a result, Goodhart’s law comes into play and the target no longer works as anticipated. When central government becomes aware of the disconnect between reported and actual performance this further reduces its trust in front-line services, and historically the response to this loss of trust has been increased reporting requirements that are more complex, more directive and more onerous. Thus, a spiral of mistrust ensues. Meanwhile the public tend to lose trust in reporting by both local services, and especially central government.

## A New Way Ahead

Another approach is needed to address this loss of trust.

One that we have advocated elsewhere^18^ involves central government, local services and the public working together to agree necessary local measures to deliver high-quality services. There is some precedent and a considerable literature regarding this approach with regard to local public service agreements introduced under New Labour in the United Kingdom in the early 2000s. In this example, a changed relationship between central and local government to a model where dialogue and negotiation set agreed focuses for improvement was associated with improved outcomes.^19-21^ While the literature suggests this new co-operative, trust-based approach was not a panacea for all ills,^19^ and successful implementation was crucial, there is much to learn from the experience, and from the wider literature of performance management in complex systems. In particular, recent work on performance management in environments that are characterised by change and uncertainty points to a shift from performance management to a learning rather than control mechanism, and the need for performance management to be more flexible and devolved.^22,23^ In the United Kingdom, the nature and spirit of the co-operative approach being reflected in actual negotiations appears key, as does a coherent and consistent narrative in central government departments to guide negotiating behaviours with local authorities in order to survive staff churn and a “regression to the mean” of traditional central command-and-control practices. ‘Working in partnership with government rather than in tension’ was identified as the result – and goal – of a successful implementation.^19^

In a public health system, especially one primarily tax funded, democracy demands that government should have the right to set high-level aims for the system, and accountability demands that progress towards these aims should be reported publicly. However, the necessary actions to best advance these aims will vary between different hospitals, services and locale. In response to this, local services need to work with their local populations to co-produce plans for local improvement aligned to the high-level objectives, including appropriate, focused measures to track progress (including accepted tools such as statistical process control, cumulative sum analysis, etc, to monitor and direct improvement). These plans should be agreed with central government, be flexible to changes in the environment (whether these changes be successful improvement or addressing emergent issues), and again progress against these measures should be publicly reported. This approach makes central government and local providers partners in delivering high-quality services.

In our view there are several advantages to this approach:

- Aims that are agreed, rather than targets that are imposed, have a greater likelihood of local professional ownership and support, and are more likely to lead to genuine, clinically and locally relevant change.
- Because of this the incentive to game measures is reduced – technical responses to discourage gaming are important and have a role, but certainly when embedded within a culture of local ownership and trust seem likely to be more effective.
- Mutually agreed aims are more likely to generate trust across the system. Without this health services will not be able to address the challenges they face in the 21st century.

In terms of the challenges noted above in the UK local public service agreement experience, the New Zealand health sector’s largely positive experience with the co-developed “System Level Measures” programme may have primed the pump for spread and scale of a truly national, co-operative, trust-based approach to setting and agreeing local contributions to national aims.^24^

Some form of monitoring of health services is now inevitable. Equally inevitable is the risk that these regimes create perverse unintended consequences such as the gaming that Tenbensel and colleagues identify. The choice open to us is whether we respond purely technically to this risk or think deeper about how monitoring and targets can strengthen systems. The latter, with particular reference to how to encourage trust between different parts of the system, is likely to be a more successful strategy.

## Ethical issues

Not applicable.

## Competing interests

Authors declare that they have no competing interests.

## Authors’ contributions

RH and CS both contributed to the drafting of the manuscript. Both authors read, reviewed and approved the final manuscript.
